# Experimental evidence for enhanced top-down control of freshwater macrophytes with nutrient enrichment

**DOI:** 10.1007/s00442-014-3047-y

**Published:** 2014-09-07

**Authors:** Elisabeth S. Bakker, Bart A. Nolet

**Affiliations:** 1 Department of Aquatic Ecology, Netherlands Institute of Ecology, Droevendaalsesteeg 10, 6700 AB Wageningen, The Netherlands; 2 Department of Animal Ecology, Netherlands Institute of Ecology, Droevendaalsesteeg 10, 6700 AB Wageningen, The Netherlands

**Keywords:** Grazing, Herbivory, Nutrient concentration, Omnivory, Palatability

## Abstract

The abundance of primary producers is controlled by bottom-up and top-down forces. Despite the fact that there is consensus that the abundance of freshwater macrophytes is strongly influenced by the availability of resources for plant growth, the importance of top-down control by vertebrate consumers is debated, because field studies yield contrasting results. We hypothesized that these bottom-up and top-down forces may interact, and that consumer impact on macrophyte abundance depends on the nutrient status of the water body. To test this hypothesis, experimental ponds with submerged vegetation containing a mixture of species were subjected to a fertilization treatment and we introduced consumers (mallard ducks, for 8 days) on half of the ponds in a full factorial design. Over the whole 66-day experiment fertilized ponds became dominated by *Elodea nuttallii* and ponds without extra nutrients by *Chara globularis*. Nutrient addition significantly increased plant N and P concentrations. There was a strong interactive effect of duck presence and pond nutrient status: macrophyte biomass was reduced (by 50 %) after the presence of the ducks on fertilized ponds, but not in the unfertilized ponds. We conclude that nutrient availability interacts with top-down control of submerged vegetation. This may be explained by higher plant palatability at higher nutrient levels, either by a higher plant nutrient concentration or by a shift towards dominance of more palatable plant species, resulting in higher consumer pressure. Including nutrient availability may offer a framework to explain part of the contrasting field observations of consumer control of macrophyte abundance.

**Electronic supplementary material:**

The online version of this article (doi:10.1007/s00442-014-3047-y) contains supplementary material, which is available to authorized users.

## Introduction

Vertebrate herbivores can strongly affect vegetation biomass both through direct consumption and through altering nutrient availability in terrestrial systems (McNaughton et al. 1997; Pastor et al. 2006; Bakker et al. 2009; Schrama et al. 2013). However, the importance of vertebrate herbivores as a factor structuring submerged vegetation is unclear (Lodge et al. 1998). Generally, the abundance of primary producers is controlled by bottom-up and top-down forces which may interact with each other in both terrestrial and aquatic systems (Shurin et al. 2006; Gruner et al. 2008). However, whereas growth and abundance of freshwater macrophytes are strongly influenced by the availability of resources for plant growth [recently reviewed in Bornette and Puijalon (2011)], the importance of top-down control by vertebrate consumers is debated because field studies yield contrasting results (Marklund et al. 2002). Herbivores can strongly reduce macrophyte abundance (Van Donk and Otte 1996; Weisner et al. 1997; Hilt 2006), but in other water bodies no effect of herbivores on macrophyte biomass could be found (Perrow et al. 1997; Marklund et al. 2002; Rip et al. 2006). The biomass density of grazers, particularly waterfowl, as well as grazer identity, may explain part of the observed variation (Wood et al. 2012a), whereas others argue that grazers only have important effects under restricted conditions of low vegetation density or in certain seasons (Marklund et al. 2002).

However, nutrient quality of the vegetation is another factor that could explain the variable results. Generally, the proportion of primary production that is removed by herbivores increases with plant nutritional quality (often expressed as foliar N concentration), both in terrestrial and aquatic systems (Cebrian and Lartigue 2004; Shurin et al. 2006). Macrophytes take up nutrients from the sediment and the water column (Carignan and Kalff 1980; Madsen and Cedergreen 2002) and plant nutrient concentration increases with nutrient availability in the water body (Cronin and Lodge 2003), making plants more attractive for consumers (Dorenbosch and Bakker 2011). Furthermore, increased nutrient availability may also lead to a shift in vegetation composition (Blindow et al. 1993; Van den Berg et al. 1999), which may in turn affect plant palatability, as macrophyte species differ in palatability to generalist consumers (Elger et al. 2004; Dorenbosch and Bakker 2011).

Although increasing nutrient availability may alter plant nutrient quality, it also modifies plant growing conditions, which will affect plant regrowth after grazing (Wise and Abrahamson 2007). Factors that limit macrophyte growth change from nutrients to light over a gradient of increasing nutrient availability (Bornette and Puijalon 2011). Macrophyte biomass initially increases with increased nutrient availability, but macrophytes eventually disappear under eutrophic conditions with phytoplankton dominance, large epiphyton loading and as a result, strong light limitation (Sand-Jensen and Borum 1991; Scheffer et al. 1993; Jeppesen et al. 2000; Hilt 2006). Therefore, macrophyte tolerance to grazing may be altered with nutrient availability (Gayet et al. 2011). Furthermore, consumers can also increase nutrient availability in the water through allochthonous nutrient input (Manny et al. 1994; Hahn et al. 2008) and through an increase in autochthonous nutrient cycling (Mitchell and Wass 1995; Vanni 2002). This may enhance plant growth in nutrient-limited systems, but not in water bodies that are already eutrophic.

To date, there have not been any controlled experiments that simultaneously investigated the effects of nutrient status of a water body and waterfowl grazing impact. We created ponds of different nutrient status through fertilization and introduced facultative herbivorous ducks (northern mallards *Anas platyrhynchos* L.) on half of the ponds. We removed the ducks after 8 days, and measured plant biomass as well as plant and water nutrient concentrations in the duck and control ponds, both immediately after the ducks were removed and 6 weeks later, to be able to measure direct and indirect effects of duck presence.

We hypothesize that:

1. Direct consumer impact is larger in nutrient-rich than nutrient-poor water bodies, which could be explained by higher plant nutritional quality.
2. Indirect consumer impact on macrophyte biomass depends on (re)growth after the presence of consumers which could be: (a) higher in nutrient-rich than nutrient-poor systems, as more nutrients for growth are available in the latter; or (b) higher in nutrient-poor than nutrient-rich systems, due to light limitation in the latter.

## Materials and methods

### Experimental ponds

We conducted the experiment in 2007 and used 20 experimental ponds out of a set of 36 which had been established in 2005 in Loenderveen, the Netherlands (52°12′N, 5°02′E); see also Bakker et al. (2010). Each pond was 1.25 m deep with 0.3 m of sediment (10:1 sand and clay mixture). The water was controlled with a standpipe and fixed at 0.5-m depth. The ponds were square shaped (with slopes of 45°) with 20 m^2^ of water surface area and 9 m^2^ of sediment surface area and held 7 m^3^ of water. The ponds contained a mixed macrophyte vegetation dominated either by *Elodea nuttallii* Planch. St John (hereafter *Elodea*) in the fertilized ponds or *Chara globularis* Thuill (hereafter *Chara*) in the ponds without extra nutrients. Other species were present in low amounts including: *Ceratophyllum demersum* L., *Myriophyllum spicatum* L., *Potamogeton pectinatus* L., *Potamogeton perfoliatus* L. and *Ranunculus circinatus* Sibth. *Elodea* is a facultative rooting species that reproduces clonally in our region, and *Chara* has rhizoids and is a spore plant which forms oogonia later in the season. Both species can take up nutrients from the sediment and the water column (Vermeer et al. 2003; Angelstein and Schubert 2008; Wüstenberg et al. 2011). Ponds were fishless and covered with nets to prevent grazing by wild waterfowl. The ponds were arranged in a grid with 1.5 m between ponds. The ponds had been cleared of remaining above sediment macrophyte biomass in winter through raking and were re-filled with fresh dephosphatised lake water in early spring (water properties of inlet water: 0.650 mg NH_4_–N L^−1^, 0.650 mg NO_3_–N L^−1^ and  0.007 mg PO_4_–P L^−1^; Waternet, unpublished data).

### Nutrient treatment

Half the ponds received weekly nutrient additions during the growing seasons in 2006 (Bakker et al. 2010) and in 2007 (60 g NH_4_NO_3_ and 15 g KH_2_PO_4_, which corresponds with 3.0 mg N L^−1^ and 0.5 mg P L^−1^, respectively) between 4 May and 20 August. These nutrient levels were chosen to simulate a eutrophic condition under which submerged macrophytes would still be able to grow (Portielje and Roijackers 1995; Van de Bund and Van Donk 2004; Bakker et al. 2010). The other half did not receive any additional nutrients. High rainfall levels in 2007 kept the water level at 0.5 m all summer.

### Grazing experiment

We used captive mallards (*Anas platyrhynchos* L.) as our model consumer species. Mallard ducks are omnivorous, but their diet consists of about 90 % plant material (Wood et al. 2012a). Mallards are able to reach a depth up to 40 cm when dabbling (Kear 2005). In a test trial the mallards readily consumed macrophytes when these were offered in feeding trays in a shallow water layer. We used only female mallards in our experiment to avoid males being distracted by nearby female presence. The experiment was approved by the Animal Experiments Committee of the Royal Netherlands Academy of Arts and Sciences (protocol CL2007.01).

Macrophytes were sampled on 15 June to determine biomass before the start of the duck experiment (see the section “Measurements” below for the sampling methods). We then selected 20 ponds of the 36 (ten with and ten without nutrient addition) which contained enough biomass to sustain mallards for at least 7 days, containing >700 g dry macrophyte standing crop per pond, based on the assumption that ducks need about 10 % of their wet body weight as dry weight food (Kear 2005), i.e. approximately 100 g dry macrophytes per mallard per day. We randomly assigned the duck treatment to five ponds of two nutrient levels. Before the ducks were introduced on the ponds, macrophyte biomass was equal among the nutrient treatments and the assigned duck treatments (two-way ANOVA, nutrient treatment, *F*_1,16_ = 0.57, *P* = 0.46; duck treatment, *F*_1,16_ = 0.003, *P* = 0.96; nutrients × duck, *F*_1,16_ = 0.052, *P* = 0.82). On 2 July 2007 mallards were introduced in ten ponds (one mallard per pond); primaries were clipped before duck release. The ponds were fenced with a low fence around the pond about 1 m from the edge and a net on top which was 1 m above the ground around the pond and 2 m high in the middle of the pond. The area around each pond consisted of tiles, without terrestrial vegetation. The ducks were not provided with additional food other than that naturally present in the ponds. We placed two floating platforms (0.5 × 0.5 m) in these ponds, of which one contained a roof to provide shelter. The ducks used the platforms for resting and defecating. Although ducks could climb out of the pond, we observed only one to do so once. Also, we found no tracks or droppings on the tiles that could indicate that ducks left the ponds when we were not looking, whereas we found a lot of these tracks on the platforms in the pond. Rain washed away most of the droppings from the platforms; where necessary we washed the platforms in the ponds daily to permit nutrients from the faeces to return to the ponds. All ducks produced droppings during the experiment on multiple days. All mallards were removed on 10 July 2007, eight days after their introduction. The length of the feeding trial was based on the amount of macrophytes in the ponds on 15 June, where the pond with the lowest macrophyte biomass was calculated to be able to sustain mallard feeding for 8 days (see above). After the mallards were removed weekly nutrient addition was continued until 20 August.

### Measurements

Macrophyte biomass was sampled on 15 June, 2 weeks before the start of the experiment, on 11 July, the day after mallards were removed and on 20 August, 6 weeks after mallard grazing. Macrophyte biomass was collected from a round metal sampler with a diameter of 0.5 m (0.2 m^2^) and 0.5 m height, which was placed randomly in each pond while avoiding duplicate sampling in time. Also, the area where the floating platforms could cast shade on the vegetation was avoided. One sample/pond per harvest date was taken, to avoid too much disturbance of the vegetation. The sampler was pressed firmly into the sediment and macrophytes were collected by hand, cleaned in the lab, sorted to species and dried at 60 °C for 4 days. Only charophytes were not sorted to the species level; *Chara globularis* was the dominant species with small amounts of *Chara vulgaris* present (Bakker et al. 2010).

The nutrient concentration in the dominant plant species was determined in subsamples taken from the biomass harvest on 11 July. *Elodea* plants were collected from all ponds and *Chara* from all unfertilized ponds and from three fertilized ponds, as it was rare in these ponds. By 20 August it had disappeared altogether from the fertilized ponds; therefore, plant material from 20 August was not further analysed. All plant material was thoroughly cleaned in the lab. The rest of each sample was dried for 3 days at 60 °C and ground (1-mm mesh). C and N concentration was determined through combustion on an element analyser (Euro EA 3000; Hekatech, Wegberg, Germany). The P concentration of the macrophytes was determined by first incinerating the ground samples for 30 min at 500 °C, followed by a 2 % persulphate digestion step in an autoclave for 30 min at 121 °C. The digested samples were analysed using a QuAAtro segmented flow analyser (Seal Analytical, Beun de Ronde, Abcoude, the Netherlands).

We collected water samples from each pond on 2 (before duck release) and 11 July (immediately after) and 20 August (6 weeks after) by taking a 200-mL sample from each pond. Chlorophyll *a* concentrations were determined using the PHYTO-PAM fluorometer (Heinz Walz, Effeltrich, Germany) (Lürling and Verschoor 2003). To measure nutrient availability in the ponds we filtered the water samples over a 0.7-µm-mesh GF/F Whatman filter and analysed them with continuous-flow analysis on an auto-analyser (Skalar Sanplus Segmented Flow Analyser; Skalar Analytical, Breda, the Netherlands) to determine PO_4_, NO_3_ and NH_4_ concentrations in the water. The pH and conductivity was measured in situ with a portable probe (340i SET, 2E30-101B02; Wissenschaftlich Technische Werkstätten, Weilheim, Germany) in the field. Light (photosynthetically active radiation) was measured with a LI-190 Quantum Sensor and LI-192 (underwater) (LI-COR Biosciences, Lincoln, NE), light availability was expressed as the percentage of light available at the bottom of the pond (50-cm depth), relative to ambient light. Alkalinity was measured in the lab by titration with 0.05 M HCl.

Epiphyton load on the plants was measured on 26 July on *Elodea* as this was the only species which occurred in all the ponds in sufficient densities. We collected three branches of *Elodea* plants (mean 0.26 ± 0.02 SE g dry weight in total) per pond, placed these in a 250-mL bottle filled with filtered (0.7-µm mesh) lake water and shook the bottle gently for 1 min following the method of Zimba and Hopson (1997). We selected pieces of 5-10 cm of fresh and green *Elodea* stems of the upper part of the shoots. *Elodea* pieces were removed, dried at 60 °C and weighed. The water samples containing the algae were filtered over washed Whatman GF/F filters, the filters were ashed for 2 h at 555 °C and algal biomass was calculated from the weight difference of the filters before and after ashing and divided by the dry weight of the *Elodea* branches in each bottle.

Because snail grazing can strongly affect epiphyton abundance (Jones et al. 2002) we counted the number of snails that were floating on the water surface as a proxy for snail abundance on 30 July. These were all *Lymnea stagnalis* L.

### Data analysis

We used a repeated-measures ANOVA to test whether consumer impact on macrophyte biomass is larger in nutrient-rich than nutrient-poor water bodies with nutrient treatment and duck presence as fixed factors and time in the experiment as repeated measure (before, immediately after, and 6 weeks after introducing ducks). The data followed a normal distribution and homogeneity of variances was obtained without transformations. We indeed found a significant interaction between nutrient treatment and duck presence as well as a three-way interaction between time of measurement, nutrient treatment and duck presence. Therefore, we further tested the impact of duck presence per date for the unfertilized and fertilized ponds using independent *t*-tests on which we applied a Bonferroni correction for multiple testing. The direct effect of ducks could be determined immediately after removal of the ducks, the indirect effect 6 weeks after introduction of the ducks. To test whether the nutrient concentrations (N and P) in *Chara* and *Elodea* plants were higher in fertilized ponds, we used a three-way ANOVA with nutrient treatment, duck presence and plant species as fixed factors. Data were log 10 transformed to obtain homogeneity in variances. There was a significant three-way interaction among the fixed factors, therefore plant nutrient concentrations were tested for each plant species separately. This revealed that there was no effect of duck presence, therefore this treatment was removed as a factor and plant nutrient concentrations were tested with two-way ANOVAs with nutrient treatment and plant species as fixed factors. To test whether water nutrient availability and light availability affected plant (re)growth, repeated-measures ANOVAs with nutrient and duck treatment as fixed factors were used. Nutrient data were log 10 transformed and light availability was square root transformed to obtain homogeneity of variance and a normal distribution of the data. Epiphyton load was tested with a Kruskall-Wallis test as the data were not normally distributed, also not after transformation. Differences among nutrient and duck treatments were tested with a multiple rank test. Snail density was tested with a two-way ANOVA after log 10 transformation with nutrient and duck presence as fixed factors. The relationship between epiphyton biomass and snail density was tested with a Spearman rank correlation.

All statistical analyses were performed in Statistica 12 (StatSoft 2013).

## Results

### Effect of duck presence on macrophyte biomass

**Fig. 1:**
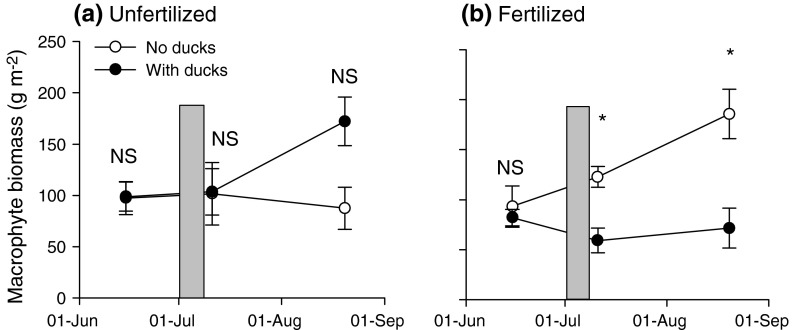
Macrophyte biomass in **a** unfertilized and **b** fertilized ponds before the ducks were introduced (15 June), immediately after the ducks had been present (11 July) and 6 weeks after ducks had been present (20 August). Ducks stayed on the ponds from 2 to 11 July (*grey bar*). *Asterisk* indicates significantly different biomasses between ponds with ducks and ponds without ducks in unfertilized and fertilized treatments (*P* < 0.05); *NS* not significant. Data are means + SE (*n* = 5). Results of the repeated-measures ANOVA and paired *t*-tests are presented in Tables 1 and 2

There was a strong interaction between nutrient treatment and duck presence on macrophyte biomass, which changed over time (Table 1; Fig. 1). Immediately after duck presence, macrophyte biomass was reduced by approximately 50 % in the fertilized ponds, whereas mallards had no measurable effect on macrophytes in the unfertilized ponds (Table 2; Fig. 1). At the end of the growing season, 6 weeks after the mallards had been present on the ponds, macrophyte biomass had increased in the fertilized ponds without ducks, whereas the fertilized vegetation where ducks had been present had not grown much during this time, resulting in significantly lower biomass in the ponds where ducks had been (Fig. 1b). In contrast, in the unfertilized ponds, if anything, macrophyte biomass had increased in the ponds where ducks had been present, but the difference in biomass between the duck treatments was not significant (Table 2; Fig. 1a).

**Table 1:** Results of repeated-measures ANOVA with nutrient treatment and duck presence as fixed factors and macrophyte biomass and water nutrient and light availability in the experimental ponds as dependent variables

	Macrophyte biomass			NO_3_ (mg L^−1^)		NH_4_ (mg L^−1^)		PO_4 _(mg L^−1^)		Light (% on bottom)	
	*df*	*F*	*P*	*F*	*P*	*F*	*P*	*F*	*P*	*F*	*P*
Nutrient treatment	1.16	0.24	0.63	90.70	<*0.001*	21.35	<*0.001*	228.07	<*0.001*	17.42	<*0.001*
Duck presence	1.16	1.14	0.30	0.22	0.64	0.88	0.36	5.71	*0.030*	0.32	0.58
Nutrients × ducks	1.16	8.52	*0.010*	5.55	0.03	3.48	0.08	3.86	0.067	0.00	0.99
Time in the experiment	2.32	7.89	*0.002*	48.93	<*0.001*	10.43	<*0.001*	14.49	<*0.001*	2.54	0.09
Time × nutrients	2.32	0.16	0.85	31.64	<*0.001*	7.76	*0.002*	0.21	0.81	0.80	0.46
Time × ducks	2.32	0.86	0.43	1.48	0.24	3.54	*0.041*	0.23	0.79	1.47	0.24
Time × nutrients × ducks	2.32	11.40	<*0.001*	0.70	0.50	1.77	0.19	0.97	0.39	0.17	0.84

Data were tested over time before ducks were released in the ponds (2 July), immediately after ducks were removed (11 July) and 6 weeks later (20 August). Significant results at *P* < 0.05 are indicated in* italic*. Differences among treatments are indicated in Fig. 1 and 3, respectively

**Table 2:** Results of independent *t*-tests comparing the macrophyte biomass in ponds with ducks and without ducks, for the unfertilized and fertilized ponds, respectively

	June		July		August	
	*t*_8_	*P*	*t*_8_	*P*	*t*_8_	*P*
No nutrients	−0.06	0.95	−0.05	0.96	−2.72	0.026
With nutrients	0.49	0.64	3.94	*0.004*	3.61	*0.007*

Data are presented in Fig. 1.* df* = 8 for all analyses. Bonferroni corrections were applied for the six comparisons made across nutrient treatments; α is then 0.008, significant values after Bonferroni correction are indicated in* italic*

In the unfertilized ponds *Chara* was the dominant species with 78–95 % abundance in the biomass samples over time (see Fig. 1, Online Resource 1). The fertilized ponds were increasingly dominated by *Elodea* with 40–99 % abundance in the biomass samples over time. The ponds where ducks were introduced tended to have initially a larger proportion of *Chara* than the ponds without ducks, but both duck treatments became strongly dominated by *Elodea* over time. No duck impact on species composition for either nutrient treatment was visible (see Online Resource 1).

### Plant nutrient concentrations

**Fig. 2a–d:**
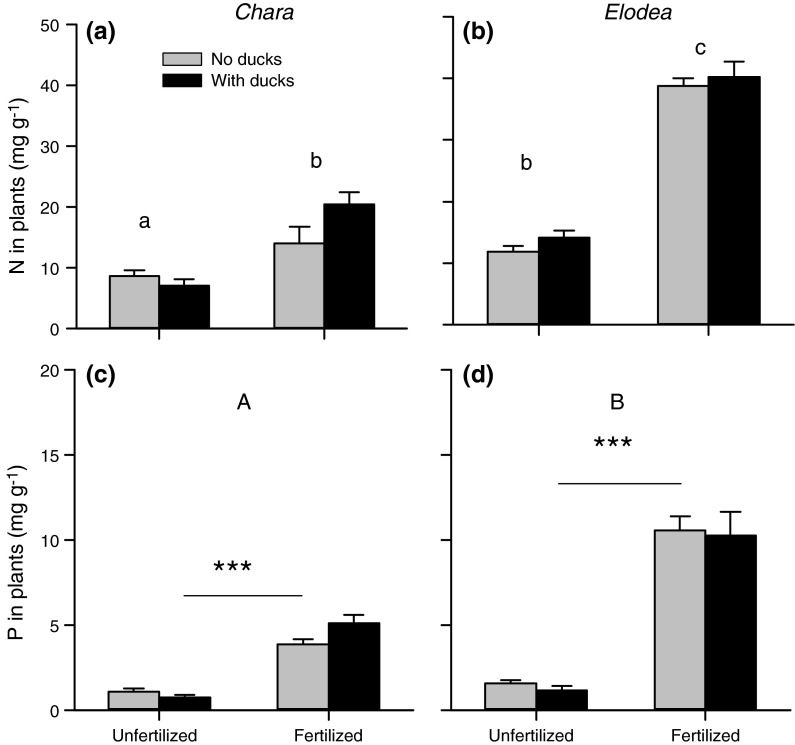
Plant nutrient concentrations in experimental ponds under different nutrient treatments and duck presence in *Chara* and *Elodea* plants. Data are means + SE (*n* = 5) and represent the values on 11 July, immediately after the ducks were removed from the ponds. *Different letters* indicate significantly different plant nutrient concentrations between nutrient treatments and plant species. Differences among duck treatments were not significant. For N concentrations there was a significant interaction between nutrient treatment and plant species, for the P concentrations only the main effects of nutrient treatment (indicated by *asterisks*) and plant species (indicated by *capital letters*) were significant. See Table 3 and “Results” for results of the statistical analyses. ***P < 0.001

Nutrient addition resulted in a doubling and tripling of N in *Chara* and *Elodea* plants, respectively, and more than four to seven times higher P concentration, respectively (Fig. 2; Table 3). There was a significant three-way interaction among nutrient treatment, duck presence and plant species on plant N concentration (Table 3). However, further testing of the effect of nutrient treatment and duck presence revealed no significant effect of duck presence on the nutrient concentrations of either plant species (see Table 1, Online Resource 1). When removing duck treatment as a factor, it became apparent that plant nutrient concentrations differed strongly among nutrient treatments and plant species. The concentration of P was higher in *Elodea* compared to *Chara* and higher in the fertilized ponds (two-way ANOVA, nutrient treatment *F*_1,31_ = 202.18, *P* < 0.001; plant species, *F*_1,31_ = 20.78, *P* < 0.001; nutrients × species, *F*_1,31_ = 3.13, *P* = 0.087). The concentration of plant N depended on the nutrient status of the pond and the plant species: *Chara* in the unfertilized ponds contained the lowest plant N concentration, whereas *Elodea* in the fertilized ponds contained the highest plant N concentration (two-way ANOVA: nutrient treatment, *F*_1,31_ = 135.43, *P* < 0.001; plant species, *F*_1,31_ = 72.59, *P* < 0.001; nutrients × species, *F*_1,31_ = 5.22, *P* = 0.029).

**Table 3:** Results of three-way ANOVA with nutrient treatment, duck presence and plant species as fixed factors and plant nutrient concentrations (N and P) as dependent variables

	N in plants (mg g^−1^)		P in plants (mg g^−1^)	
	*F*	*P*	*F*	*P*
Nutrient treatment	158.60	*<0.001*	225.42	*<0.001*
Duck presence	2.06	0.16	1.49	0.23
Plant species	85.36	*<0.001*	23.80	*<0.001*
Nutrients × ducks	2.42	0.13	4.50	*0.043*
Nutrients × species	5.70	*0.024*	3.10	0.09
Ducks × species	0.01	0.93	0.37	0.55
Nutrients × ducks × species	6.34	*0.018*	0.49	0.49

Significant results at *P* < 0.05 are indicated in* italic*

### Water nutrients and light availability

**Fig. 3a–d:**
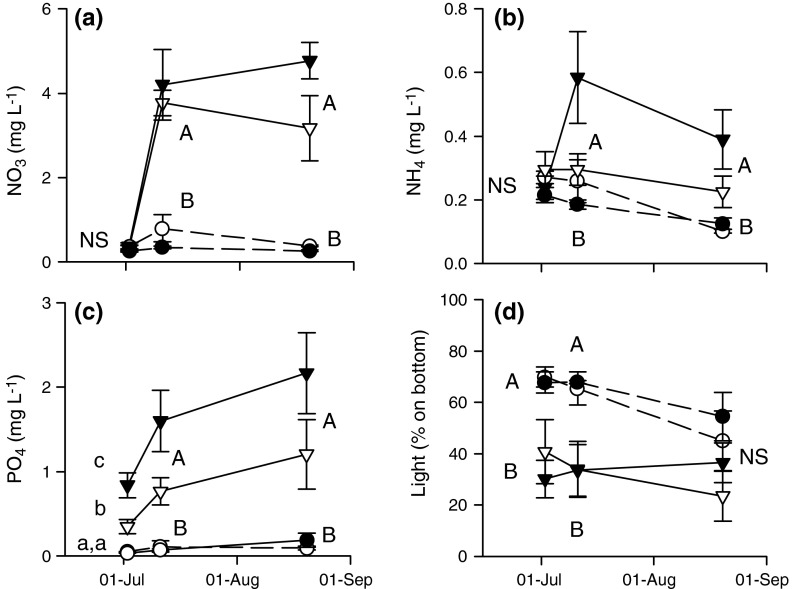
Nutrient and light availability in the experimental ponds. Data were collected before (2 July), immediately after (11 July) and 6 weeks (20 August) after the presence of ducks on the ponds. **a** NO_3_, **b** NH_4_, **c** PO_4_ and **d** light availability at the bottom of the ponds relative to ambient light. Data are means + SE (*n* = 5). Data were tested with repeated-measures ANOVAs; see Table 1 for results. *Different letters* indicate statistically different treatments for each parameter tested per date (two-way ANOVA, followed by post hoc Tukey test if a significant interaction between nutrient and duck treatment was present; *P* < 0.016, as a result of Bonferroni correction for three dates tested per parameter). *Capital letters* indicate a significant main effect of nutrient treatment; *small letters* indicate significant differences among nutrient and duck treatments as a result of a significant interaction between nutrient and duck treatments. *Dashed line* Unfertilized, *solid line* fertilized, *open symbols* no ducks, *filled symbols* with ducks

Water nutrient concentrations were higher in the fertilized ponds, whereas there were no significant effects of duck presence at any of the sampling dates (Table 1; Fig. 3a–c). Before ducks were introduced, the fertilized ponds with an assigned duck treatment turned out to have a higher PO_4_ concentration than the fertilized ponds which no ducks were assigned to. This difference was no longer significant after ducks had been present on the ponds (Fig. 3c). Almost twice as much light reached the bottom of the unfertilized compared to the fertilized ponds (Fig. 3d; Table 1). Light availability in the fertilized ponds was still quite high, about 20–40 % of the ambient light. The effect of ducks was not significant and there was no trend in time (Table 1). The chlorophyll *a* concentration in the ponds ranged from 7 to 1,334 µg L^−1^ and was on average 107 ± 29 µg L^−1^ (mean ± SE) with no trend in time, pH in the ponds was 9.4 ± 0.05 with no trend in time, conductivity decreased from 141 ± 4 at the first sampling date to 124 ± 3 µS cm^−1^ in August and alkalinity decreased from 1.52 ± 0.05 to 0.88 ± 0.04 mEq L^−1^ during the experiment. There were no significant effects of duck or nutrient treatments on chlorophyll a concentration due to large variation in chlorophyll *a* concentrations within treatments, and no effects on pH, conductivity and alkalinity either (data not shown).

### Epiphyton and snails

**Fig. 4:**
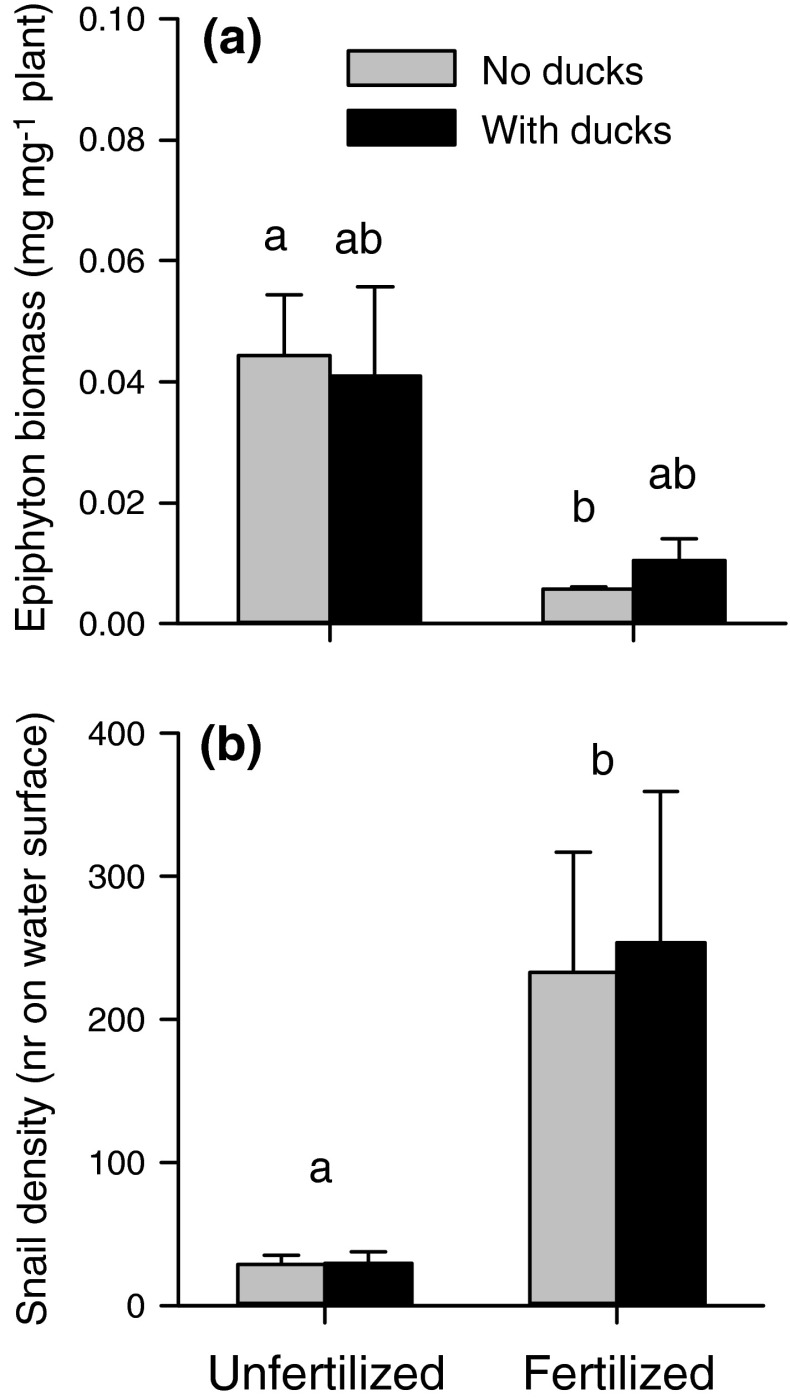
**a** Epiphyton biomass on *Elodea* (mg dry weight mg^−1^ *Elodea* dry weight) at the end of July (*n* = 5). *Different letters* indicate statistically different amounts of epiphyton on *Elodea* (Kruskall-Wallis test) across the nutrient and duck treatments. **b** Snail presence expressed as the number of floating *Lymnea* snails on the water surface in each pond at the end of July. *Different letters* indicate statistically different numbers of snails between the nutrient treatments; there were no differences between the duck treatments (two-way ANOVA). See “Results” for statistical tests

Epiphyton biomass was four times higher on *Elodea* plants in the ponds without versus those with added nutrients (Fig. 4a). This difference was significant between the treatments where no ducks had been, whereas the ponds where ducks had been were intermediate in epiphyton biomass (Kruskall-Wallis test, *H*_3,20_ = 9.56, *P* = 0.023). The density of floating snails was more than sevenfold higher in the fertilized compared to the unfertilized ponds (Fig. 4b; ANOVA, *F*_1,16_ = 10.12, *P* = 0.006), whereas there was no effect of duck presence (*F*_1,16_ = 0.02, *P* = 0.90) and no significant interaction (*F*_1,16_ = 0.01, *P* = 0.92).

There was a negative relationship between snail density and epiphyton biomass (Spearman rank correlation: *r* = −0.55, *P* = 0.013).

## Discussion

We found evidence that consumer impact on macrophytes varies with pond nutrient status. Consumers had no effect on macrophyte biomass under nutrient-poor conditions, whereas they strongly reduced macrophyte biomass in fertilized ponds. In contrast to field studies, waterfowl species and density were controlled in our ponds and the differences thus depend on the properties of the ponds. We hypothesized that differences in plant nutritional quality or plant growth conditions may explain increased consumer impact on macrophyte biomass under nutrient-rich conditions. Below we discuss the evidence for the hypothesized mechanisms that may explain this result.

### Plant quality, nutrient addition and grazing pressure

Plant nutrient concentration was significantly affected by the nutrient treatments. Both *Chara* and *Elodea* plants contained a two to sevenfold higher N and P concentration when grown in fertilized ponds compared to unfertilized ponds. Therefore, we found support for our first hypothesis that increased nutrient availability in the environment resulted in higher grazing pressure, which coincided with higher nutrient concentration of the plants. Alternatively, these results can also be explained by a species-specific difference in palatability of *Chara* and *Elodea*, if *Elodea* is more palatable irrespective of its nutrient concentration. However, mallards did not seem to feed preferentially on *Elodea*, which occurred amongst the dominant *Chara* vegetation in the unfertilized ponds at up to 20 % of the total biomass. The N and P concentrations were generally low in *Chara* and *Elodea* under nutrient-poor conditions and clearly highest in *Elodea* in the fertilized ponds. This suggests that the nutrient-poor conditions were important in the lack of mallard feeding on macrophytes, apart from a possible general preference for *Elodea* over *Chara*. Separate feeding trials to determine duck preference for these macrophyte species showed that both species are palatable for mallard ducks (Ahmad, Bakker and Klaassen, unpublished data). Our study is representative for generalist consumers, whereas specialist feeders, such as red-crested pochard (*Netta rufina* Pallas), which feed particularly on charophytes, may induce different effects (e.g. Matuszak et al. (2012)). In our study the effect of plant species and pond nutrient status cannot be fully separated and the relative importance of plant species and plant nutrient concentrations in determining grazing pressure thus remains to be investigated in more detail. Furthermore, whereas nutrient addition led to higher plant nutrient concentrations in several experiments with macrophytes (Cronin and Lodge 2003; Dorenbosch and Bakker 2011), it should be noted that the nutrient concentrations found in plant tissues do not necessarily always reflect nutrient concentrations found in the water (Casey and Downing 1976).

An increase in plant nutrient concentrations after nutrient addition and a concomitant increase in grazing pressure have been found across ecosystems. Nutrient addition increased the N concentration or decreased the C:N ratio in macrophytes, which enhanced consumption by fish (Dorenbosch and Bakker 2011), in salt marsh plants, which became more attractive for geese (Bos et al. 2005; Stahl et al. 2006), and in grassland plants leading to higher grazing pressure by rabbits (Bakker et al. 2005). Similarly, sea turtles (Christianen et al. 2012) and ungulates (Van der Wal et al. 2003) are attracted to fertilized plants with increased N concentrations. Plant N concentration is generally found to correlate positively with herbivore consumption (Cebrian and Lartigue 2004). For herbivorous ducks and geese, plant quality such as N concentration is an important determinant of foraging decisions (Durant et al. 2004), and the strong increase in geese and swan numbers in north-western Europe has been at least partly attributed to the increased use of fertilizer on agricultural lands (Van Eerden et al. 2005). Therefore, nutrient availability and plant nutritional quality may be an important parameter to consider when predicting grazing pressure on submerged macrophytes.

### Herbivory and omnivory in aquatic vertebrates

No significant removal of vegetation was found in the unfertilized ponds, suggesting that no macrophytes were consumed by the ducks. The ducks may have fasted to a certain extent, but they also ate, as droppings were observed for all ducks in both nutrient treatments during the 8 days that the ducks stayed on the ponds. Because the droppings were returned to the ponds, in order to study nutrient cycling, no diet data are available, but most likely, the mallards ate macro-invertebrates, as they are facultative herbivorous species (Kear 2005; Wood et al. 2012a). Macro-invertebrates are abundant in the ponds (Declerck et al. 2011) and are a good food source for many waterfowl species, particularly the smaller species (Wood et al. 2012a). Most waterfowl species that consume macrophytes are omnivorous (Wood et al. 2012a) and they can shift to alternative prey when macrophytes are an unprofitable food source. We observed such a diet switch of facultative herbivorous fish in a different experiment in the same ponds (Dorenbosch and Bakker 2012): they prefer macroinvertebrates over macrophytes, but increase macrophyte consumption when plants are fertilized (Dorenbosch and Bakker 2011). In the wild, ducks on water bodies of low nutrient status may either move to other water bodies or shift their diet towards macroinvertebrates.

Apart from plant quality, the foraging costs may determine whether macrophytes are being consumed by waterfowl. Elevated costs of foraging can make macrophytes unattractive food resources, and cause waterfowl to shift to alternative food resources (Wood et al. 2013). Waterfowl which have to reach macrophytes by upending have greater foraging costs than when feeding on food resources just below the surface (Guillemain et al. 2000; Nolet et al. 2006). As *Elodea* can be a canopy-forming species and *Chara* species remain at the bottom, foraging on *Elodea* may be less costly than on *Chara* sp. As our ponds were shallow with 50-cm water depth there was little difference in plant height and therefore the difference in foraging effort seemed small, but we cannot entirely exclude that *Elodea* may have been somewhat less costly to access for the ducks.

### Plant (re)growth after consumer presence

The longer term impact of consumers on plants depends on the recovery of the plants after being grazed and the indirect effects of consumer presence, including alterations of nutrient availability. Our second hypothesis was partly supported: the macrophytes responded differently to consumer presence in the nutrient treatments. The plants in the unfertilized ponds seemed not to be grazed and therefore there was no recovery 6 weeks after duck presence either. In the fertilized ponds, we observed a lack of regrowth of *Elodea* after duck presence, whereas in the ponds without ducks *Elodea* biomass had increased by 51 % after 6 weeks.

Plant regrowth after grazing is affected by nutrient and light availability (Hawkes and Sullivan 2001; Wise and Abrahamson 2007). The reduced amount of *Elodea* after grazing may have lost competition for nutrients from phytoplankton and epiphyton which possibly induced light limitation for macrophyte (re)growth (Sand-Jensen and Borum 1991; Hilt 2006), but we cannot test this because algal density (chlorophyll *a* concentration) and epiphyton load were not elevated under fertilized conditions. In the case of epiphyton, there was even less in the fertilized ponds, probably due to the grazing pressure by snails (Jones et al. 2002; Bakker et al. 2013). Also, light availability in the fertilized ponds was still rather high (minimum 20 % of ambient light on the bottom of the shallow pond) considering that *Elodea* sp. plants can still grow under very low light availability, also after being cut from the mother plant (Abernethy et al. 1996; Barrat-Segretain 2004). Possibly, snail grazing prevented recovery of *Elodea* after duck grazing, as snails can inhibit sprouting of plants and thus regeneration, when present in high enough densities (Elger et al. 2007). Furthermore, grazing by invertebrates and waterfowl has been shown to induce reallocation of resources to belowground plant parts and subsequent early senescence in above-sediment plant material in several macrophyte species (Hidding et al. 2009; Miler and Straile 2010). This may, therefore, also be an explanation for limited above-sediment recovery of grazed plants. Whereas herbivores can change the nutrient availability by importing nutrients from elsewhere (Kitchell et al. 1999; Hahn et al. 2008), through the consumption of plants and return of nutrients via faeces (Vanni 2002), we did not find evidence of enhanced nutrient availability as the nutrient concentration was not elevated in the water column nor in the macrophytes after the presence of the ducks in both nutrient treatments. Therefore, lower plant regrowth after grazing in the fertilized ponds confirms the pattern of our hypothesis 2b, which seems to be not due to abiotic factors, as we hypothesized, but possibly to biotic factors of snail grazing or plant reallocation of resources.

### Comparison with the field

The two dominant macrophyte species in our ponds are also frequently found in the field where alkaline oligotrophic-to-mesotrophic waters are commonly dominated by charophytes (Van de Bund and Van Donk 2004; Rip et al. 2006; Ibelings et al. 2007), whereas mesotrophic-to-eutrophic waters are often dominated by *Elodea* sp. (Van Donk and Otte 1996; Perrow et al. 1997; Van de Haterd and Ter Heerdt 2007). Most studies that measured grazing impact on *Chara*-dominated vegetation found no significant effect of grazing on plant biomass during the summer (Van den Berg 2001; Rip et al. 2006; Hidding et al. 2010); but see Matuszak et al. (2012). In two eutrophic lakes dominated by *Elodea* sp., herbivores significantly reduced plant biomass (Van Donk and Otte 1996; Van de Haterd and Ter Heerdt 2007). The results of our pond study could explain the differences observed in these field studies, but we should keep in mind that in the field the density and species of consumers may differ among water bodies, which can change the impact on the macrophytes (Wood et al. 2012a, b). Currently few field studies of consumer control of aquatic macrophytes consider or report plant species identity or nutrient availability. We conclude that including water nutrient status and identity of the dominant plant species in the analysis of consumer control of macrophyte biomass may provide a framework with which to understand and predict top-down control in aquatic benthic systems.

## Electronic supplementary material

Below is the link to the electronic supplementary material.

Supplementary material 1 (DOCX 202 kb)
