# Supplementary material for: Experimental evidence for enhanced top-down control of freshwater macrophytes with nutrient enrichment
Source: Oecologia. 2014 Sep 7;176(3):825–36. doi: 10.1007/s00442-014-3047-y (PMC4207960; doi:10.1007/s00442-014-3047-y)

**Electronic supplementary material**

**Experimental evidence for enhanced top-down control of freshwater macrophytes with nutrient enrichment**

Elisabeth S. Bakker^1,3^ and Bart A. Nolet^2^.

^1^Department of Aquatic Ecology, Netherlands Institute of Ecology, Droevendaalsesteeg 10, NL-6700 AB Wageningen, The Netherlands.

^2^Department of Animal Ecology, Netherlands Institute of Ecology, Droevendaalsesteeg 10, NL-6700 AB Wageningen, The Netherlands.

^3^corresponding author: Department of Aquatic Ecology, Netherlands Institute of Ecology, Droevendaalsesteeg 10, NL-6700 AB Wageningen, The Netherlands. Phone: +31 317 473400. E-mail: [l.bakker@nioo.knaw.nl](mailto:l.bakker@nioo.knaw.nl)

Corresponding author: Elisabeth S. Bakker, Department of Aquatic Ecology, Netherlands Institute of Ecology, Droevendaalsesteeg 10, NL-6700 AB Wageningen, The Netherlands. Phone: +31 317 473400. E-mail: [l.bakker@nioo.knaw.nl](mailto:l.bakker@nioo.knaw.nl)

**Table 1.** Results of Two-way ANOVA with nutrient treatment and duck presence as fixed factor and plant N and P concentration as dependent variables for *Chara* and *Elodea* plants. Significant values at the level P<0.05 are indicated in bold.

|  | N in plants (mg g^-1^) | | P in plants (mg g^-1^) | |
| --- | --- | --- | --- | --- |
| *Chara* | F_1,11_ | *P* | F_1,11_ | *P* |
| Nutrient treatment | 25.40 | **<0.001** | 70.89 | **<0.001** |
| Duck presence | 0.57 | 0.47 | 0.15 | 0.71 |
| Nutrients x Ducks | 4.05 | 0.07 | 3.21 | 0.10 |
| *Elodea* | F_1,16_ |  | F_1,16_ |  |
| Nutrient treatment | 281.27 | **<0.001** | 176.19 | **<0.001** |
| Duck presence | 2.27 | 0.16 | 2.10 | 0.17 |
| Nutrients x Ducks | 1.16 | 0.30 | 1.26 | 0.28 |

**Fig 1** Plant species composition in the unfertilized (a) and fertilized (b) ponds where ducks were allowed and the ponds without ducks (*n*=5). Before ducks were released (15 June), immediately after duck presence (11 July) and six weeks after ducks were on the ponds (20 August). *Elodea*: *Elodea nuttallii*, *Chara* spp. *Chara globularis* with very small amounts of *Chara vulgaris*. Other species included *Ceratophyllum demersum* L., *Myriophyllum spicatum* L., *Potamogeton pectinatus* L., *Potamogeton perfoliatus* L. and *Ranunculus circinatus* Sibth. Flab – floating algal beds.


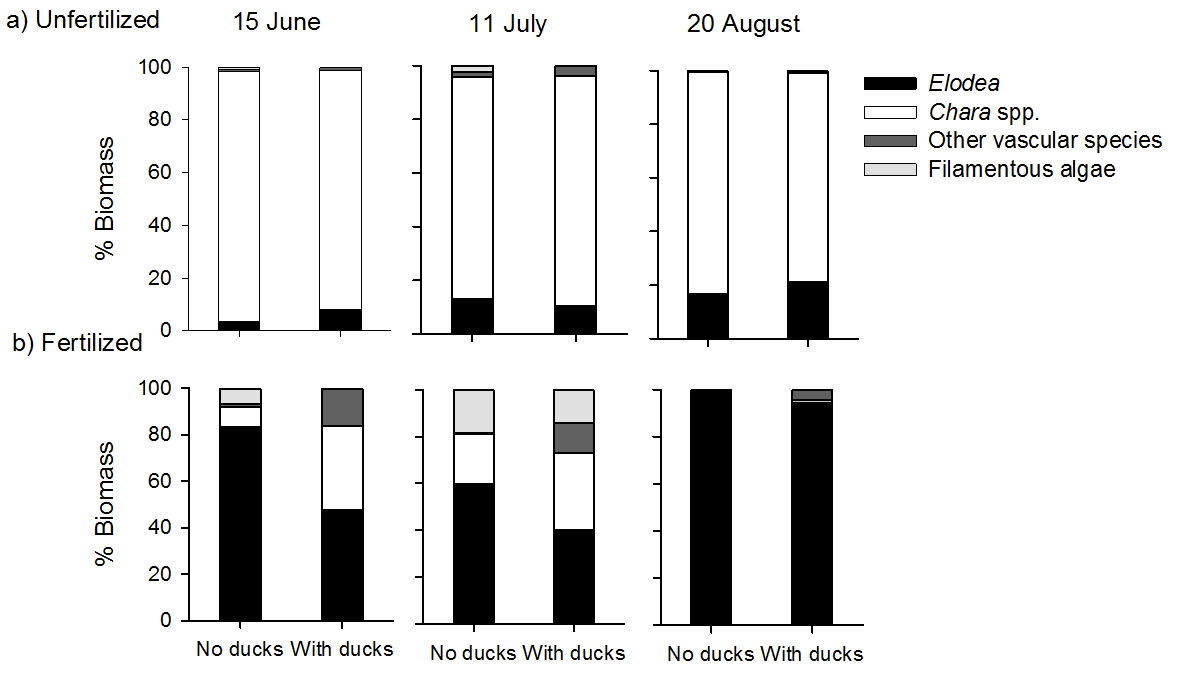

Supplement: Supplementary file 1 — Supplementary material 1 (DOCX 202 kb) [file 442_2014_3047_MOESM1_ESM.docx]
